# Fast processing of microscopic images using object-based extended depth of field

**DOI:** 10.1186/s12859-016-1373-2

**Published:** 2016-12-22

**Authors:** Apichart Intarapanich, Saowaluck Kaewkamnerd, Montri Pannarut, Philip J. Shaw, Sissades Tongsima

**Affiliations:** 1 National Electronics and Computer Technology Center, National Science and Technology Development Agency, Thailand Science Park, Pathum Thani, Thailand; 2 National Center for Genetic Engineering and Biotechnology, National Science and Technology Development Agency, Thailand Science Park, Pathum Thani, Thailand

## Abstract

**Background:**

Microscopic analysis requires that foreground objects of interest, e.g. cells, are in focus. In a typical microscopic specimen, the foreground objects may lie on different depths of field necessitating capture of multiple images taken at different focal planes. The extended depth of field (EDoF) technique is a computational method for merging images from different depths of field into a composite image with all foreground objects in focus. Composite images generated by EDoF can be applied in automated image processing and pattern recognition systems. However, current algorithms for EDoF are computationally intensive and impractical, especially for applications such as medical diagnosis where rapid sample turnaround is important. Since foreground objects typically constitute a minor part of an image, the EDoF technique could be made to work much faster if only foreground regions are processed to make the composite image. We propose a novel algorithm called object-based extended depths of field (OEDoF) to address this issue.

**Methods:**

The OEDoF algorithm consists of four major modules: 1) color conversion, 2) object region identification, 3) good contrast pixel identification and 4) detail merging. First, the algorithm employs color conversion to enhance contrast followed by identification of foreground pixels. A composite image is constructed using only these foreground pixels, which dramatically reduces the computational time.

**Results:**

We used 250 images obtained from 45 specimens of confirmed malaria infections to test our proposed algorithm. The resulting composite images with all in-focus objects were produced using the proposed OEDoF algorithm. We measured the performance of OEDoF in terms of image clarity (quality) and processing time. The features of interest selected by the OEDoF algorithm are comparable in quality with equivalent regions in images processed by the state-of-the-art complex wavelet EDoF algorithm; however, OEDoF required four times less processing time.

**Conclusions:**

This work presents a modification of the extended depth of field approach for efficiently enhancing microscopic images. This selective object processing scheme used in OEDoF can significantly reduce the overall processing time while maintaining the clarity of important image features. The empirical results from parasite-infected red cell images revealed that our proposed method efficiently and effectively produced in-focus composite images. With the speed improvement of OEDoF, this proposed algorithm is suitable for processing large numbers of microscope images, e.g., as required for medical diagnosis.

## Background

Microscopic imaging is a widely used technique in life science in which two-dimensional images are acquired from three-dimensional cellular specimens. An important skill in microscopy is adjusting the focus in order to obtain clear images of biological features. A typical biological specimen will have several different features of interest that are located on different depths of field (DoF). Automated image acquisition can be used to acquire stacking images from different DoFs. The combined images can be processed using an algorithm to create a composite image that captures all features in-focus. This type of image is known as an extended depth of field (EDoF) image. Several algorithms have been proposed to generate EDoF images based on selecting regions with high saliency [1]. The research efforts in [2–5] focused on improving the EDoF algorithm using pixel domain and transform domain methods. In 2004, Forster and colleagues [5] proposed a complex-valued wavelet transformation that can accurately measure the weight of each detail information from input images. Other computational methods for obtaining high-quality EDoF images have been proposed that involve sophisticated selection criteria based on geometric transformation techniques such as the ridgelet transform [6], wedgelet transform [7], contourlet transforms [8] and curvelet transform [9]. Although all of these approaches are capable of generating high-quality EDoF images, the computational complexity of these algorithms grows quadratically with the number of pixels in each image. This high computational demand means that it is impractical to generate EDoF images from multiple specimens. In some applications of microscopy, for example medical diagnosis, sample turnaround time is very important. A more computationally efficient method for acquiring EDoF images could form the basis of a rapid automated image acquisition and diagnosis platform.

In a typical microscopic specimen, the features of biological interest are likely to be spread sparsely and unevenly over the field of view. Therefore, digital images of microscopic specimens will comprise mostly background and a minority of foreground pixels. If an image processing algorithm can identify foreground objects and selectively process only the pixels within these objects, the overall image processing time will be dramatically reduced. Microscopy-based medical diagnosis typical requires detailed observations of samples involving many fields of view, since features of interest, e.g., parasites, are sparsely distributed. Therefore, to confirm diagnosis, standard operating procedure requires processing of many images. For example, in diagnosis of malaria infection, greater than 100 fields of view must be examined [10]. In this work, we present a novel image fusion technique based on the extended depth of field concept, called object-based extended depth of field (OEDoF). The proposed OEDoF workflow constructs the final EDoF composite image by focusing only on specific regions that contain objects of interest and thus dramatically cuts down the computational time. This algorithm is implemented as an ImageJ plugin and was used to reconstruct composite images from multiple optical sectioned images of biological specimens obtained from a malaria diagnostic laboratory. The implemented OEDoF software and the images used in this paper are publicly available for downloading from http://www4a.biotec.or.th/GI/tools/oedof.

## Methods

The data used to test the algorithm comprised 250 images obtained from 45 thick film slides prepared from malaria-infected blood specimens. The images were obtained using an in-house automated image-capturing platform, and were published previously in [11]. No new samples were collected for this study, and thus no ethical approval is required. The resolution of these images was set to 928 × 616 pixels with 24 bit depth. The computational processing of the stack images to create composite EDoF images was conducted on a MacBook Pro notebook (Apple Inc., USA) equipped with an Intel core 2 duo 2.4Ghz processor and 8 GB random access memory.

### Object-based extended depth of field (OEDoF) algorithm

**Fig. 1:**
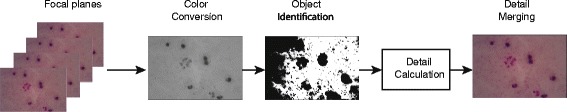
Object-based Extended Depth of Field (OEDoF) framework. The OEDoF technique consists of four major procedures, namely color conversion, region segmentation, detail calculation and detail merging

The reconstruction process used to create an EDoF image is computationally intensive as all the picture elements from multiple stacking images must be checked in order to select only the in-focus objects to be merged at the end. In order to obtain a high-quality EDoF image suitable for accurate biological interpretation with less computational effort, we propose object-based extended depth of field (OEDoF). The OEDoF approach comprises four main modules, namely color conversion, object identification, detail calculation and detail merging (Fig. 1).

### Preprocessing: color conversion

Microscopic images generally have low contrast, which creates difficulty in differentiating foreground objects from the background. Thus, we enhance the underlying contrast by projecting all significant information of the RGB color space into a new color space that is suitable for further analysis. A color conversion module is employed for this task that uses independent component analysis [12] to extract the significant color space information. Eq 1 represents the *C* color space calculation in which the weighting coefficients (*a*_*1*_ *, a*_*2*_ *, a*_*3*_) are derived from the covariance matrix.

1 $$ C={a}_1+{a}_2G+{a}_3B $$

To calculate the weighting coefficients, an RGB covariance matrix M that represents all pixels is constructed. This matrix allows adaptive weighting in accordance with the characteristics of each individual image as follows:

2 $$ M=\frac{1}{n-1}{\displaystyle \sum_{i=1}^n}\left({X}_i-\overline{X}\right) $$

where *X*_*i*_ is each color component in RGB format of the *i* ^th^ pixel and *x̅* represents the mean of the RGB components and *n* is total number of pixels. The elements along the diagonal of the covariance matrix represent the weighting coefficients. The image with the highest variance among those obtained from the focus stack is selected as a baseline image for reconstructing the composite all in-focus image during the last step of OEDoF.

### Object region identification

**Fig. 2:**
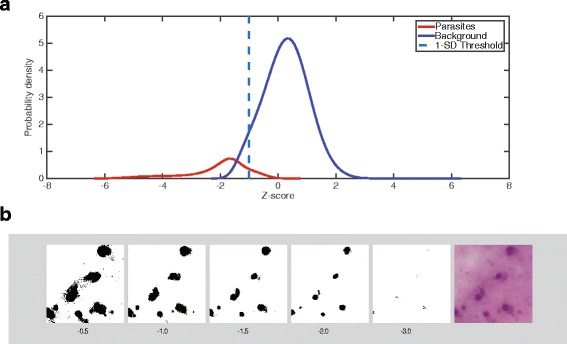
Assignment of foreground pixels using Z-score thresholding **a**. Kernel density plots were made of Z-scores for pixels obtained from 21 images of malaria thick film specimens. Z-scores were calculated from the distribution of pixel brightness values from the combined 21 images (average brightness = 0.5640; SD = 0.0849). Foreground objects were assigned by manual inspection of the images and were delimited by drawing circles around them. All pixels within the boundaries of these objects were assigned as foreground pixels, and all others outside were assigned as background. The Z-score threshold of −1 (indicated by the dashed line) was incorporated into the algorithm for automatic assignment of foreground pixels for EDoF composite image construction. **b** Representative specimen showing foreground pixel assignment using different Z-score thresholds. The original image captured from the Giemsa-stained specimen is shown on the far right. The adjacent panels show the background masked areas in white and the foreground in black using Z-score thresholds of −0.5, −1, −1.5, and −2

The major novel feature of the OEDoF algorithm is object identification, in which only regions of potential interest are processed further for the sake of computational efficiency. Identification of foreground objects first requires assigning pixels in each focus stack image into background and foreground. To accomplish this task, the distribution of pixel brightness values in each image is determined. We empirically determined that a Z-score threshold of −1 can be used to assign foreground pixels, which is fast to calculate and consistent across different sample images. To obtain this threshold, the combined pixel brightness distribution was determined from 21 randomly selected thick blood film images. Foreground objects (cells) were assigned manually by drawing circles around them. The pixel brightness values were extracted from the foreground objects, while all other pixels were assigned as background. Z-scores were calculated for each pixel using the combined distribution mean and standard deviation values. Kernel density plots of foreground and background pixel Z-scores are shown in Fig. 2A. From this plot, it can be seen that the majority of foreground pixels have Z-scores less than −1. The foreground objects are darker than the background as the cells are stained with Giemsa. The results of using different Z-score thresholds for assigning foreground object pixels in a representative image are shown in Fig. 2B. In this example, all cells are assigned as foreground objects when using a Z-score threshold of −1. The process of assigning foreground and background pixels is performed on all images in the focus stack. The union of foreground pixels from all depths of field is used for the next step of identifying foreground objects.

### Good contrast pixel identification

The aim of this process is to identify good contrast pixels to be incorporated into the final composite EDoF image. This is because pixels with higher contrast are likely to be more in-focus than the ones with lower contrast. For each pixel, we calculate the value representing the pixel contrast by comparing with the eight adjacent pixel neighbors and the corresponding pixels on the top and bottom layer depths. We adopted the 3D Sobel kernel operator [13] to compute the underlying contrast value for each pixel.

The 3 × 3 Sobel kernels *G*_*x*_ performs the vertical mask on the left and right columns of the target pixel (the middle column contains zeros). The 3 × 3 Sobel kernels *G*_*y*_ performs the horizontal mask on the top and bottom row of the target pixel (the middle row contains zeros).

3 $$ \begin{array}{cc}\hfill {G}_x=\left[\begin{array}{ccc}\hfill +1\hfill & \hfill 0\hfill & \hfill -1\hfill \\ {}\hfill +2\hfill & \hfill 0\hfill & \hfill -2\hfill \\ {}\hfill +1\hfill & \hfill 0\hfill & \hfill -1\hfill \end{array}\right]\hfill & \hfill {G}_y=\left[\begin{array}{ccc}\hfill +1\hfill & \hfill +2\hfill & \hfill +1\hfill \\ {}\hfill 0\hfill & \hfill 0\hfill & \hfill 0\hfill \\ {}\hfill -1\hfill & \hfill -2\hfill & \hfill -1\hfill \end{array}\right]\hfill \end{array} $$

We added the third 3 × 3 Sobel kernel *G*_*z*_ to mask the neighboring pixels above and below the target pixels as follows:

4 $$ \begin{array}{ccc}\hfill {G}_z(1)=\left[\begin{array}{ccc}\hfill -1\hfill & \hfill -2\hfill & \hfill -1\hfill \\ {}\hfill -2\hfill & \hfill -4\hfill & \hfill -2\hfill \\ {}\hfill -1\hfill & \hfill -2\hfill & \hfill -1\hfill \end{array}\right]\hfill & \hfill {G}_z(0)=\left[\begin{array}{ccc}\hfill 0\hfill & \hfill 0\hfill & \hfill 0\hfill \\ {}\hfill 0\hfill & \hfill 0\hfill & \hfill 0\hfill \\ {}\hfill 0\hfill & \hfill 0\hfill & \hfill 0\hfill \end{array}\right]\hfill & \hfill {G}_z(1)=\left[\begin{array}{ccc}\hfill +1\hfill & \hfill +2\hfill & \hfill +1\hfill \\ {}\hfill +2\hfill & \hfill +4\hfill & \hfill +2\hfill \\ {}\hfill +1\hfill & \hfill +2\hfill & \hfill +1\hfill \end{array}\right]\hfill \end{array} $$

Finally, the Gradient magnitude *G* is calculated from *G*_*x*_ , *G*_*y*_ and *G*_*z*_ as follows:

5 $$ G=\sqrt{G_I^2+{G}_y^2}+{G}_z^2 $$

Note that the above Sobel filtering step only operates on the foreground pixels; thus the selection of pixels with high contrast can be done quickly. The pixels of greatest contrast from all layers are used to reconstruct the final composite image in the next step.

### Detail merging for image reconstruction

We perform the image reconstruction by combining the pixels of greatest contrast from individual depth fields together. In particular, the pixels from R, G and B components representing the highest gradient magnitude *G* are used to construct the final composite image. To complete the image reconstruction, we replace all pixels from the baseline image previously selected during the image preprocessing (color conversion) with the highest contrast pixels identified by the algorithm.

**Fig. 3:**
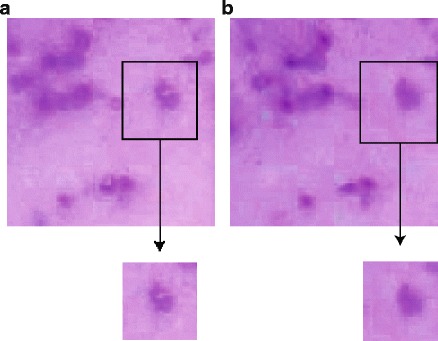
Comparison of composite images before and after color consistency correction Images before (left panel) and after (right panel) color correction are shown. The shading aberration (see the close-up boxes) can be corrected using 5x5 filtering mask

However, combining these pixels (which may come from different depths) in the final composite image may introduce color inconsistency for some objects, as pixels from different focal planes could have slightly different color shades. To address this shading aberration, color consistency correction is required. A 5 × 5 pixel grid is positioned in foreground regions of the composite image. For each of the 25 pixels in this window, the original focal plane is recorded. A majority-voting rule is used to identify the focal plane that contributes the most high-contrast pixels to the composite image within the 5 × 5 grid. The pixel from this focal plane is used to replace the pixel at the center of the grid in the final composite image. The 5 × 5 grid is repositioned by sliding one pixel and the process repeated until all foreground pixels have been analyzed (Fig. 3).

### Software package

We created a software tool to generate a composite focused image using the proposed OEDoF technique. The tool is implemented as an ImageJ plugin using Java language. This plugin can be downloaded from http://www4a.biotec.or.th/GI/tools/oedof. The instruction on how to use the OEDoF plugin in ImageJ as well as the sample images used in our study are also available from this website.

## Results and Discussion

**Fig. 4:**
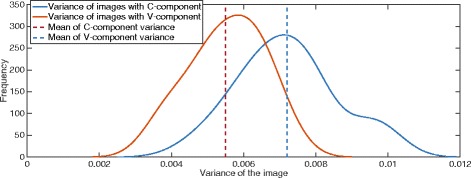
The comparison of V-component and C-component variances. A total of 21 RGB images of malaria infected red blood cell specimens were converted and we compared the V-component variance (red curve) of HSV color space with the C-component variance (blue curve) of the proposed *C* color space. The mean of C-component variance is higher than the mean of V-component variance, which implies that the C-component has more contrast information

The performance of the OEDoF algorithm was tested and compared with the complex wavelet approach [5], which we considered as the “gold standard” in its ability to generate high-quality composite images. The algorithms were compared in terms of image quality and computational time to generate the EDoF image. We used images of thick-film specimens obtained from malaria-infected cases for testing the algorithm. Microscopic examination is widely used for malaria diagnosis, but low accuracy and slow sample turnaround can be a problem as an automated image acquisition and processing system is not available. Fast and accurate image processing algorithms could help diagnosis of malaria and other diseases requiring microscopic analysis. Rapid and accurate image processing first requires optimization of image contrast. We found that the newly created *C* color space provides better contrast information from the RGB than the HSV (Hue, Saturation and Value) color format that is popularly used in many image processing applications. To demonstrate this, we converted 21 RGB images of malaria infected specimens (10 *Plasmodium falciparum* and 11 *Plasmodium vivax* infected specimens) of dimensions 4752 × 3168 pixels and compared the V-component variance (red curve) of HSV color space against the C-component variance (blue curve) of the proposed *C* color space (Fig. 4). The mean of C-component variance (blue dashed line) is higher than the mean of V-component variance (red dashed line), which implies that C-component has more contrast information.

### On image quality

**Fig. 5:**
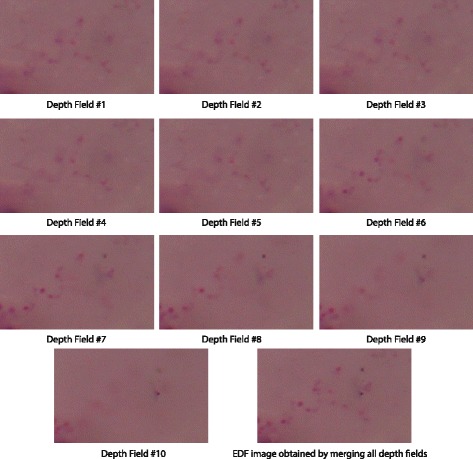
The images of each depth of field and the composite image Ten images of the same specimen taken at different depths and the composite image generated by the OEDoF algorithm are shown. The composite image shows in-focus objects located at different depth fields

**Fig. 6:**
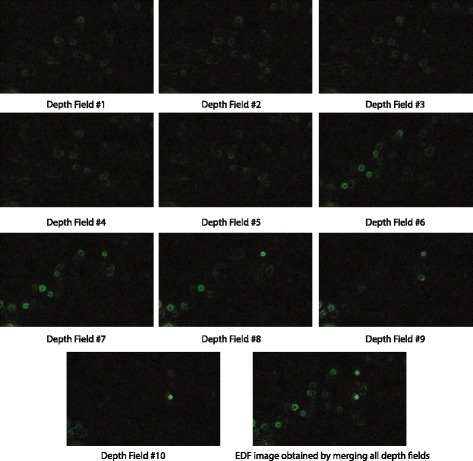
Edge detection on multiple images from different depths. To show that the OEDoF composite image can enhance the performance of image analysis, edge detection was performed using the ImageJ Find Edges command on all images shown in Fig. 5A. The corresponding images with detected edges are shown in false green color. It is clear that the OEDoF composite image provides more edge information than any individual image from different depth fields

**Fig. 7:**
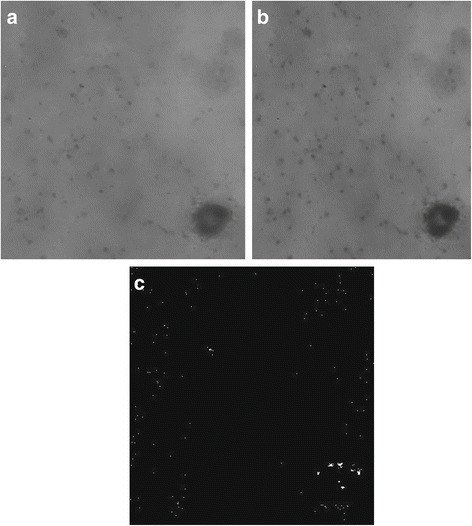
Comparison of the composite images generated by OEDoF and complex wavelet. **a** The composite image generated by OEDoF. **b** The composite image generated by the complex wavelet approach. **c** The differences of the two composite images

Next, multiple images from different depths were used to construct composite images. Ten images of the same specimen taken at different depths and the composite image generated by the OEDoF algorithm are shown in Fig. 5. The composite image shows in-focus objects located at different depth fields. To show that the OEDoF composite image can enhance the performance of image analysis, edge detection was performed on all images shown in Fig. 5. Edge detection is an important process for object identification, and the OEDoF composite image provides more edge information than any individual image from different depth fields (Fig. 6). The composite image produced by OEDoF was compared with that produced by the complex wavelet approach [5]. The OEDoF image is comparable to the complex wavelet composite image in terms of clarity due to the focus enhancement of all foreground objects (Fig. 7A, B). To demonstrate the small differences between the OEDoF and complex wavelet composite images, the pixel intensities of the complex wavelet image were subtracted from the intensities of the corresponding pixels in the OEDoF image to construct a *diff* (*i*, *j*) plot (Fig. 7C). The different pixels (white spots) account for 5.4% of the total pixels in the OEDoF composite image, which are scattered sparsely around the background and foreground areas.

### On processing time

Forty specimens were randomly selected for assessing image algorithm processing speeds. We expected the OEDoF to perform markedly faster than the state-of-the-art complex wavelet approach, since OEDoF processes only object regions, which generally make up less than half of the total image pixels. As expected, the average processing time for OEDoF was much shorter than that of complex wavelet (OEDoF average = 0.47 seconds, sd = 0.01; complex wavelet average = 1.98 seconds, sd = 0.03). Next, we tested how well the OEDoF algorithm could perform using fewer depths of field to construct composite images, since the time taken to capture images is important for applications such as medical diagnosis. Five specimens were randomly selected and a total of 10 image stacks were used from each. OEDoF composite images were constructed from 3, 4, 5, 6, 7, 8, 9, and 10 depths of field in descending order along the z-axis. To test how using fewer depths of field affects composite image quality, the acutance (*A*_*c*_) or measure of image clarity [14] of the composite images was computed using Eq. 6.

6 $$ {A}_c=\frac{1}{mn}{\displaystyle \sum_{i=0}^m}{\displaystyle \sum_{j=0}^n}\sqrt{G_I^2\left(i,j\right)+{G}_y^2\left(i,j\right)} $$

Where *m* and *n* are the image dimensions and *i*, *j* are pixel positions in *x* and *y* directions. *G*_*x*_ and *G*_*y*_ are the 3 × 3 Sobel horizontal and vertical kernels, respectively.

**Fig. 8:**
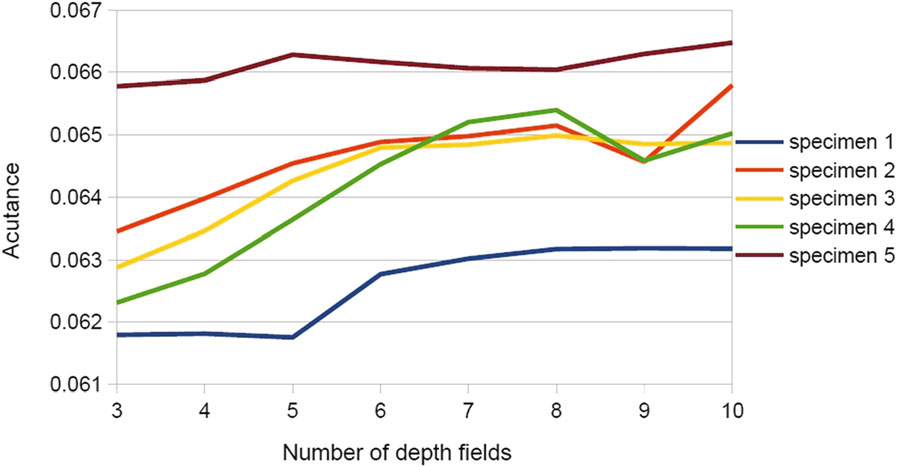
The acutance plot of composite images generated by different number of depth fields. Five specimens were randomly selected and a total of 10 image stacks were obtained from each. OEDoF composite images were constructed from 3, 4, 5, 6, 7, 8, 9, and 10 depths of field in descending order along the z-axis

As expected, *A*_*c*_ increases with the number of depth fields that are used to construct composite images. The optimal number of depth fields, i.e. the fewest needed to make a composite image with maximum acutance appears to vary from specimen to specimen (Fig. 8). However, the loss of composite image quality using fewer depths of field is not that great, and using as few as three depth fields could be adequate for downstream image processing and pattern recognition tasks.

## Conclusions

The OEDoF algorithm can create composite images with foreground objects in focus that are comparable in quality to the state-of-the-art complex wavelet EDoF algorithm, but with four-fold faster processing time. The greater computational efficiency of OEDoF is achieved by selectively processing pixels from object regions only instead of the entire image. Although some image quality is sacrificed for speed, the composite images produced by OEDoF retain foreground details sufficient for downstream biomedical image processing, in applications such as counting infected cells, differentiating malaria species, etc. Furthermore, the threshold used to identify foreground objects will depend on the contrast, which will vary depending upon the type of specimens being examined. We identified a suitable threshold for thick film specimens of malaria cases; however, other biomedical specimens, e.g., histological specimens may require a different threshold. The proposed technique should also work well with microscopic images obtained from most stained biological specimens in which backgrounds are brighter than object regions. The marked improvement in image processing time is particularly important for medical diagnosis where rapid turnaround is required. For example, microscopy-based malaria diagnosis requires at least 100 images per specimen [10]. The four-fold reduction in time using OEDoF technique could translate to improved diagnosis and treatment of diseases.
